# PReS-FINAL-2055: Is there a necessity for patients with JIA to wear orthopedic insoles?

**DOI:** 10.1186/1546-0096-11-S2-P68

**Published:** 2013-12-05

**Authors:** F Kreuzpointner, M Hartmann, A Schwirtz, JP Haas

**Affiliations:** 1 Biomechanics in sport, Technische Universität München, Munich, Germany; 2 Deutsches Zentrum für Kinder-und Jugendrheumatologie, Garmisch Partenkirchen, Germany

## Introduction

It is often discussed whether patients with juvenile idiopathic arthritis (JIA) should wear orthopedic insoles or not. The functions of insoles are to minimize pain or to assist foot deformities. JIA often goes along with foot impairments [1,2] describe different deviation and deformities which can develop of various pattern of foot joint involvements, like pes valgoplanus or hallux rigidus.

## Objectives

The aim of the study was to analyze the peak plantar pressure distribution of a well described cohort of JIA patients with an active symmetrical ankle joint arthritis and no history of foot involvement. This setting enables to study joint loadings on healthy tissue (foot joints) due to arthritis in a superior joint (ankle joint).

## Methods

Twenty two patients (14.4 ± 4.1a, 153.5 ± 12.5 cm, 46.7 ± 14.4 kg) with a symmetrical ankle joint arthritis with the subtypes sero-negative polyarticular JIA (n = 16) and systemic JIA (n = 6) and a cohort of healthy subjects (n = 15, 11.0 ± 2.0a, 147.0 ± 13.0 cm, 38.1 ± 9.9 kg) are included in this retrospective study design. Each subject had to walk five times on each side over a four sensors/cm^2 ^pressure distribution plate (Emed, Munich, Germany). For analysis the foot was divided into eleven regions of interests.

## Results

This abstract shows the loading of each metatarsal-head (MH1(medial) - MH5 (lateral)) (Tab.1). Patients have statistical significant higher peak pressure values in the forefoot except under MH3. Further on patients have a deviating pressure distribution along the transversal arch with the highest loads under MH5. (Table 1)

**Table 1:** shows the difference in peak pressure distribution between patients and controls within the metatarsal-phalangeal-joints 1-5.

[kpa] mean±SD		MH1	MH2	MH3	MH4	MH5
Patients	Left	**231.0 ± 130.3**	**333.2 ± 234.3**	293.1 ± 176.3	**324.1 ± 220.8**	**410.3 ± 362.7**

Control group	Left	**136.8 ± 36.6**	**206.7 ± 63.9**	212.3 ± 75.2	**165.7 ± 68.1**	**141.2 ± 103.3**

Patients	Right	**257.8 ± 167.6**	**376.1 ± 324.8**	291.4 ± 208.5	**281.5 ± 184.8**	**390.8 ± 376.3**

Control group	Right	**154.0 ± 43.0**	**216.5 ± 67.6**	211.8 ± 61.0	**163.2 ± 48.3**	**120.2 ± 77.9**

Numbers in bolt are statistical significant (*p *< 0.05, student's t-test).

## Conclusion

The patients included in this study suffer from an active ankle joint arthritis. They have significant higher joint loading under healthy tissue of the foot in comparison to the controls. This might be a reason for prescribing orthopedic insoles during a period of an active arthritis. The lateral shift of the peak pressure distribution within the patients in the transversal arch indicates that it is important to control the foot function and pressure distribution not only in patients with a history of foot impairment. Orthopedic insoles might be a valuable therapeutic treatment to protect healthy tissue during a period of active arthritis.

## Disclosure of interest

None declared.
